# Discovering hidden relationships between renal diseases and regulated genes through 3D network visualizations

**DOI:** 10.1186/1756-0500-3-296

**Published:** 2010-11-11

**Authors:** Suresh K Bhavnani, Arunkumaar Ganesan, Theodore Hall, Eric Maslowski, Felix Eichinger, Sebastian Martini, Paul Saxman, Gowtham Bellala, Matthias Kretzler

**Affiliations:** 1 Center for Computational Medicine & Bioinformatics, 2017 Palmer Commons Bldg., 100 Washtenaw Avenue, Ann Arbor, MI 48109-2218, USA; 2 Michigan Institute for Clinical & Health Research, 24 Frank Lloyd Wright Dr., Domino's Farm, Ann Arbor, MI 48106-0421. USA; 3 Electrical Engineering and Computer Science, University of Michigan, 2260 Hayward, Ann Arbor, MI 48109-2121, USA; 4 UM3D Lab, Digital Media Commons, University of Michigan, 2281 Bonisteel Blvd., Ann Arbor, MI 48109-0738, USA; 5 Department of Internal Medicine, Division of Nephrology, University of Michigan Medical School, 1150 W. Medical Centre Drive, MSRB2, SPC 5676 Ann Arbor, MI 48109-5676, USA; 6 Current address: Institute for Translational Sciences, University of Texas Medical Branch, 301 University Blvd. Galveston, TX 77555-0129, USA

## Abstract

**Background:**

In a recent study, two-dimensional (2D) network layouts were used to visualize and quantitatively analyze the relationship between chronic renal diseases and regulated genes. The results revealed complex relationships between disease type, gene specificity, and gene regulation type, which led to important insights about the underlying biological pathways. Here we describe an attempt to extend our understanding of these complex relationships by reanalyzing the data using three-dimensional (3D) network layouts, displayed through 2D and 3D viewing methods.

**Findings:**

The 3D network layout (displayed through the 3D viewing method) revealed that genes implicated in many diseases (non-specific genes) tended to be predominantly down-regulated, whereas genes regulated in a few diseases (disease-specific genes) tended to be up-regulated. This new global relationship was quantitatively validated through comparison to 1000 random permutations of networks of the same size and distribution. Our new finding appeared to be the result of using specific features of the 3D viewing method to analyze the 3D renal network.

**Conclusions:**

The global relationship between gene regulation and gene specificity is the first clue from human studies that there exist common mechanisms across several renal diseases, which suggest hypotheses for the underlying mechanisms. Furthermore, the study suggests hypotheses for why the 3D visualization helped to make salient a new regularity that was difficult to detect in 2D. Future research that tests these hypotheses should enable a more systematic understanding of when and how to use 3D network visualizations to reveal complex regularities in biological networks.

## Findings

### Introduction

Several studies have analyzed how diseases such as breast cancer [1-3] and leukemia [4] are similar or different at the molecular level. Such studies have led to improvements in the classification of diseases [5,6], and targeted treatment options [2,4,7]. However, little is known about how combinations of chronic renal diseases are similar or different at the molecular level.

**Figure 1:**
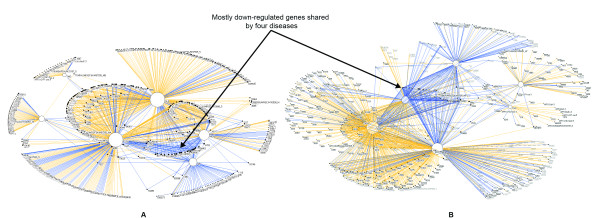
**2D and 3D Layout of the Renal Network.** The renal bipartite network laid out by the Fruchterman-Reingold [18] algorithm in 2D (A), and in 3D (B) showing the relationship between 7 renal diseases (white nodes), and 747 genes (black nodes). The size of each node is proportional to the number of its connected edges so diseases with many genes have large nodes, and diseases with few genes have smaller nodes. A yellow edge represents that the connected gene is up-regulated, and a blue edge represents that the connected gene is down-regulated. The 3D network layout enables more spreading out of nodes (as shown by the greater area used by the down-regulated genes in the centre of the 3D network), but also tends to occlude node labels.

To address this lack of understanding, in a recent study [8] we used 2D network visualizations and quantitative analyses to understand how 747 mRNA transcripts (henceforth referred to as genes) were regulated across 7 chronic renal diseases. Figure 1A shows the network that we generated and analyzed. Here the nodes represent diseases or genes, and an edge between them represents either an up or down regulation. The size of the nodes is proportional to the number of edges connected to them (node degree). Therefore large disease nodes (high degree) have relatively many associated genes, whereas small disease nodes (low degree) have few associated genes.

The network analysis revealed three unexpected regularities, with possibly important domain implications. (1) Many genes were associated with a single disease (disease-specific genes) and fewer genes were associated with many diseases (non-specific genes). This regularity related to *gene degree *resulted in a right skewed gene degree distribution. The many disease-specific genes suggested that the current classification (based on similarities in morphology, pathophysiology, or serology) is reflective of specific molecular mechanisms, whereas the few non-specific genes indicate the presence of one or more common mechanisms activated in chronic kidney disease. (2) There were unexpected combinations of renal diseases, genes, and regulation type. For example, there were four renal diseases (SLE, FSGS, MGN, IgAN) that shared many genes, which tended to be down-regulated. This regularity related to *disease type *and *regulation type *suggested the presence of a shared molecular mechanism between two different classes of diseases: mostly inflammatory (SLE, IgAN) and mostly non-inflammatory diseases (FSGS, MGN). (3) There was uniform concordance in gene regulation (genes were either up or down regulated across diseases). This regularity related to *regulation type *suggested that the shared mechanisms have identical effects on genes regulation, and could in the future help to identify molecular diagnostic markers.

Our network analysis therefore revealed complex and unexpected relationships between gene degree, disease type, and regulation type, which led to important insights about chronic renal diseases. Subsequent analyses confirmed that these relationships were unlikely to have occurred by chance. (1) A comparison to 1000 random networks of the same size (number of nodes and edges), but with edges randomly assigned to pairs of nodes, revealed a low probability (p < .05) of the observed gene degree distribution occurring by chance. (2) A comparison to 1000 random networks of the same size, and gene and disease degree distribution (to ensure the same number of high degree genes and diseases) revealed a low probability (p < .001) of 4 diseases having mostly down-regulated genes occurring by chance. (3) A comparison to 1000 random networks of the same size, degree distributions, and proportion of up and down regulated genes revealed that there was a low probability (p < .001) of the observed 100% concordance in gene regulation occurring by chance.

Although we used quantitative methods to analyze each of the above relationships, our main insights about the disease-gene relationships emerged from visually inspecting the 2D network layout. However it is well known [9] that such layouts are limited compared to laying out the same network in 3D. For example, nodes that are densely packed close to each other in 2D can be spread out in 3D to help the analyst detect more complex regularities. We therefore explored if we could deepen our understanding of genes regulated in renal diseases by using 3D network layouts.

While 3D appeared intuitive and appealing, several studies within the information visualization and cognition communities have reported conflicting results of its value in comprehending information [10]. For example, recent cognitive research has shown that users performing network-based tasks on 3D networks through stereoscopic visualizations outperform users who have access only to 2D visualizations of the same data [9]. However, others argue that the added cost of generating and learning how to analyze 3D networks far outweighs the expected benefits, and point to alternate 2D representations for networks that could potentially achieve the same results [11]. Within the bioinformatics community, most of the published literature on 3D has focused on building tools (e.g.,[12,13]). Therefore, there is currently neither consensus on the value of 3D to analyze complex datasets, nor consensus on how best to use such visualizations to analyze biological networks. Furthermore, to the best of our knowledge, none of the studies have demonstrated the value of 3D visualizations to reveal novel insights.

We therefore decided to use 3D network visualizations to reanalyze the 2D network of renal diseases and genes that we had recently published. Our primary goal was to explore if we could discover new patterns in the data that were missed in our earlier 2D network analysis. Our secondary goal was to develop hypotheses related to the pros and cons of using 3D network visualizations to analyze biological networks.

## Method

To deepen our understanding of what we already understood about chronic renal diseases and genes, we posed the following research question: *What is the relationship between chronic renal diseases, gene regulation, and gene specificity?*

To address our research question, we made critical decisions regarding data selection, data representation, data viewing, and data analysis.

### Data Selection

We conducted a secondary analysis on a dataset of 747 genes differentially regulated in 7 renal diseases. Transcriptomic data were obtained from 106 patients with one of seven chronic renal diseases, and were compared to biopsies from healthy kidney donors (control). (Please see the original study [8] for a description of the gene expression analysis, and criteria for determining up and down gene regulation.)

### Data Representation

Networks have become ubiquitous in the analysis and discovery of a wide range of molecular phenomena such as gene regulation [14], disease-gene associations [15], and disease-protein associations [16]. A network (also referred to as a graph in mathematics) consists of a set of points or nodes, joined in pairs by lines or edges; nodes represent one or more types of entities (e.g., diseases or genes). Edges between the nodes represent a specific relationship between the entities (e.g., a disease is significantly correlated with a gene). Figure 1A shows a bipartite 2D network [17] (where edges exist only between two different types of entities) of diseases and their implicated genes that we analyzed in our earlier study [8].

Networks enable multiple variables to be visually represented in the same representation thereby enabling the discovery of complex relationships. For example, the network in Figure 1A visually represents the number of genes associated to a disease by proportionally sizing the diameter of the disease nodes (large disease nodes have many implicated genes, and small disease nodes have only a few); the type of gene regulation is represented by the colour of the edges that connect a disease and gene node (a yellow edge represents that the connected gene is up-regulated, and a blue edge represents that the connected gene is down-regulated). Furthermore, the network representation enables the layout of the nodes in Euclidean space using powerful force-directed algorithms that pull together nodes that share the same neighbours, and push apart nodes which do not. Such algorithms often result in layouts where the relative distances between the nodes are meaningful in revealing complex patterns in the network. For example, the network in Figure 1A was laid out using the Fruchterman-Reingold [18] (FR) algorithm in Pajek version 1.23, which resulted in genes that were shared by many diseases to be pulled to the centre of the network, and genes that were specific to a disease to be pushed to the periphery of the network. These advantages make networks useful and versatile to represent and analyze a wide range of complex relationships in biological datasets.

As shown in Figure 1B, the above network was also reconstructed by using the 3D version of the FR algorithm. The third dimension enables the algorithm to lay out the nodes such that edge crossings are reduced, a feature that is commonly considered desirable to enhance the readability of networks [9,10]. For each node, the algorithm outputs x, y and z coordinates, which are used to create the 3D network.

### Data Viewing

The 3D network was viewed using two different viewing methods:

#### 2D Viewing and Navigation

The 3D network was viewed in 2D on a computer screen using the Pajek viewer. Similar to a Virtual Reality Modelling Language (VRML) viewer, the Pajek viewer enables the network to be rotated horizontally and vertically, and to be zoomed in and out.

#### 3D Viewing and Navigation

**Figure 2:**
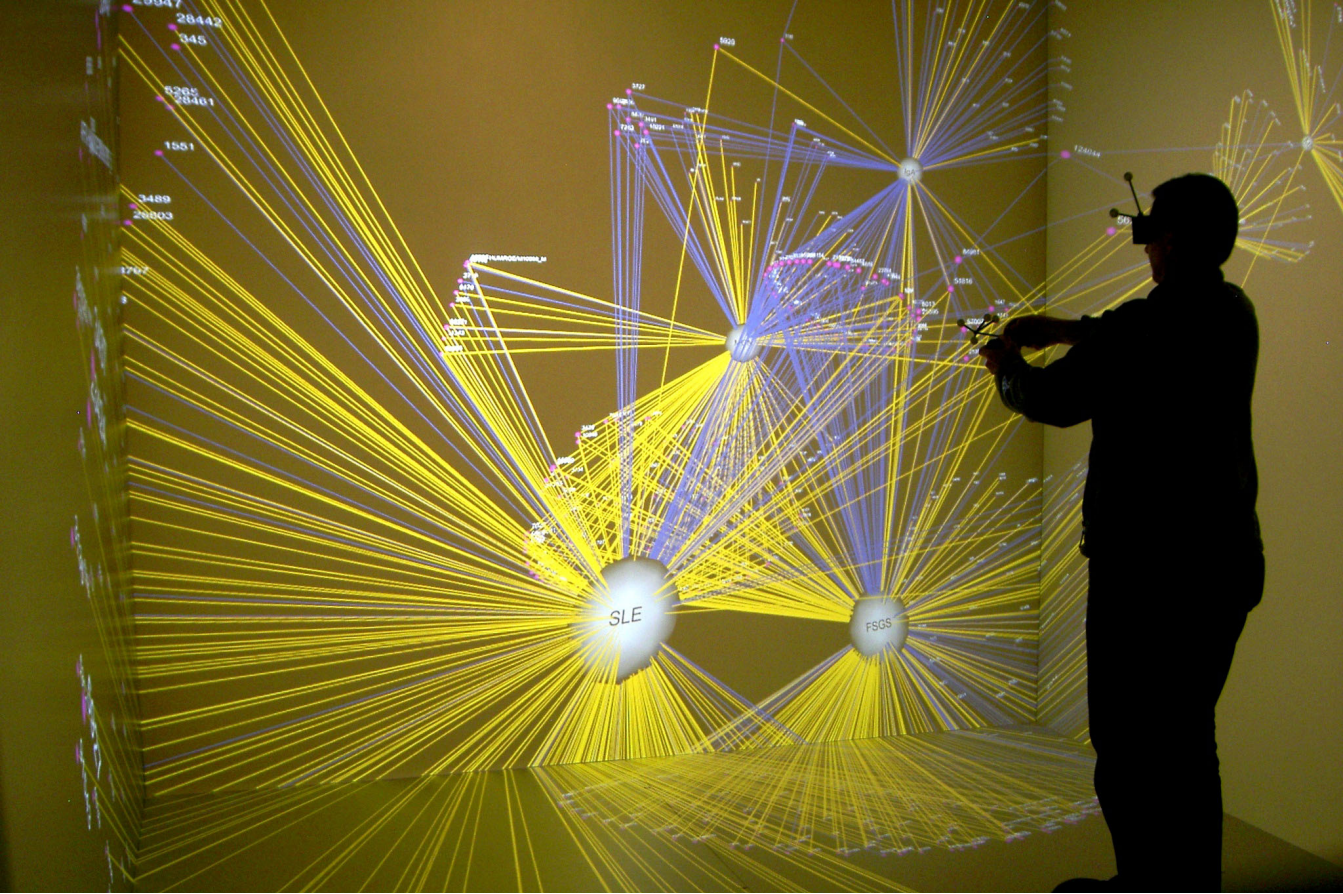
**The 3D Renal Network Viewed Inside the CAVE.** The 3D network being visualized in the CAVE through the use of stereoscopic glasses with motion sensors, and hand-held controls for rotating, and zooming in and out of the image. For clarity, the stereoscopic double image of each element has been turned off. The white nodes represent diseases, the purple nodes represent genes, and the yellow and blue edges represent up and down regulation respectively. The high magnification, wide field of view, and stereoscopic display helped to reveal the relationship between gene node degree (shown by the size of the purple gene nodes) and regulation type (shown by the blue and yellow edges that connect the gene and disease nodes).

As shown in Figure 2, the 3D network was also viewed in a 3D immersive environment called the Cave Automatic Virtual Environment (CAVE) [19]. The goals of this environment are to create the illusion that the viewer is looking at a physical object in perspective, and to enable the viewer to navigate around those virtual objects. These goals are achieved through five standard CAVE functionalities: (1) **Stereoscopic visualization **which is achieved by projecting separate left and right eye images of each object that are combined for the viewer with special eye glasses. This provides an illusion of the network objects as volumes in 3D space. (2) **Magnification **of the network enabling greater visibility of node and edge properties. (3) **Wide field-of-view **achieved by projection of the images on the inside of four surfaces (left, right, front, and floor) of a 10'x10'x10' room. This enables the viewer to see objects with a full natural field-of-view, in addition to the option of increasing this field by turning the head to look at objects above, below, sideways and behind. (4) **Motion sensors **on the eye glasses worn by the viewer enabling the viewer to walk within the CAVE, which responds by automatically detecting the location of the viewer and adjusting the perspective for the image that is being viewed. (5) **Hand-held controls **which enables the viewer to zoom, and rotate the network.

To visualize our 3D network in the CAVE, we created a program that translated the 3D output from Pajek, into one that can be processed by the CAVE system. This was done by using the OpenSG API in C++, a standard approach for CAVE applications.

### Data Analysis

We analyzed the 3D networks using the following two steps:

#### 1. Visual Analysis

The networks in both viewing modes (2D and 3D CAVE) were visually analyzed using the navigation features each provided with the goal of detecting visual patterns in the data. In each case the network was not altered in any way such as by removing or highlighting nodes. Instead, the network was viewed as a whole to identify patterns such as node clusters, and patterns in edge colouring.

#### 2. Quantitative Analysis

To obtain a precise understanding of the observed visual patterns, they were quantitatively analyzed. Because we identified a new pattern involving gene node degree and regulation type, we plotted a curve whose X-axis represented the gene degrees, and whose Y-axis represented the proportion of down-regulated edges for each degree. We then compared this curve to the same curve generated from 1000 random networks where we preserved the number of disease and gene nodes, their degree (number of edges connected to a node) distribution, and the number of up and down regulated edges. However, we randomly labelled the edges as being either up or down regulated. To test whether the pattern we observed could have occurred randomly, we determined 99.9% confidence intervals around each point on the curve.

## Results

In the introduction, we briefly presented our prior results that were based on 2D network analysis. The following are the new results based on 3D network analysis.

### 3D Network Layout Viewed in 2D

We visually analyzed the 3D network layout using the Pajek viewer on a desktop computer screen, and rotated the network in an attempt to look for regularities. Figure 1B shows the network rotated horizontally. These actions revealed three important artefacts of the 3D layout viewed in 2D. (1) Several of the nodes were occluded by edges located in front of them, and their labels were not visible. For example, the disease node SLE to the far left is occluded with edges in front, and its label is not visible. (2) The disease nodes were not in the same location as in the 2D layout, causing us to become disoriented mainly because we had over-learned the location of the disease nodes in the 2D layout. (3) Continuous rotation of the network enabled us to perceive the 3D nature of the layout. However it was difficult to read the node labels that were moving across the screen, and further contributed to our disorientation. Furthermore, when we stopped rotating the network, the 3D effect was lost due to lack of parallax (close objects moving faster compared to distant objects), and the network became partially un-readable due to edge crossings and node occlusions. (4) Zooming into the network led to additional confusion as it was difficult to comprehend the relationship between the zoomed in elements, and the entire network.

Despite the disorientation caused by viewing the 3D layout in 2D, we did confirm global regularities that we had identified in the 2D layout. There indeed was uniform concordance in gene regulation, and four of the diseases did have many down-regulated genes as shown by the mass of blue edges. However due to the disorientation and occlusions, we were unable to identify any new regularities.

### 3D Network Layout Viewed in the CAVE

#### Visual Analysis

We first analyzed the network in the CAVE by zooming out to see the entire network. Next, as shown in Figure 2, we used the hand-held controls to zoom into the network towards the centre to inspect the high density of down-regulated genes (blue edges) connected to the four diseases. The image in Figure 2 obviously can neither replicate the illusion of the network being a solid object provided by the stereoscopic effect, nor convey the immersive experience of being inside the network. We will therefore attempt to describe in words to the best of our ability the visual experience and our findings.

Our navigation of the 3D network inside the CAVE led to three key observations. (1) The large screens and zooming enabled us to look at the gene and disease nodes at a higher magnification compared to what is possible on a desktop or laptop screen. (2) Despite the zooming into the network, we were still able to see the rest of the network by merely turning our head. Therefore, we could perceive details in the network such as the labels and the node sizes, while simultaneously seeing the rest of the network, a phenomenon referred to as focus plus context [20]. Achieving a similar level of focus plus context is not possible on a typical desktop screen. (3) The stereoscopic effect enabled a group of genes with degree 4 and their edges to "pop-out" making them easily distinguishable from other nodes and edges. Therefore there was no need to rotate the network to distinguish nodes and edges from those behind or in front of them.

The above visual effects led to three new insights:

1. Because of the magnification of the network visualization in the CAVE, the gene nodes that had mostly blue edges (down-regulated) were clearly larger in size (high degree) compared to most other gene nodes. These differences in node size were not easily perceptible when viewing the network in 2D in a smaller viewing area. This relationship became even more salient when we noticed two even larger gene nodes of degree five that were both down-regulated.

2. The above relationship between high degree genes and down-regulation was inverted when we moved our head to look at genes that were smaller in size (low degree) as they were mostly connected to yellow edges (up-regulated). Therefore our earlier analysis, which revealed the relationship between *disease type *and *regulation type *(four diseases had mostly down-regulated genes), failed to reveal the current relationship between *gene degree *and *regulation type *(high degree genes were mostly down-regulated, and low degree genes were mostly up-regulated).

3. The high magnification and stereoscopic effect in the CAVE, in addition to the discovery of the above pattern, also helped to quickly identify 11 high degree genes that did not fit the above trend. Each of these genes was up-regulated as shown by their yellow edges in Figure 2.

#### Quantitative Analysis

**Figure 3:**
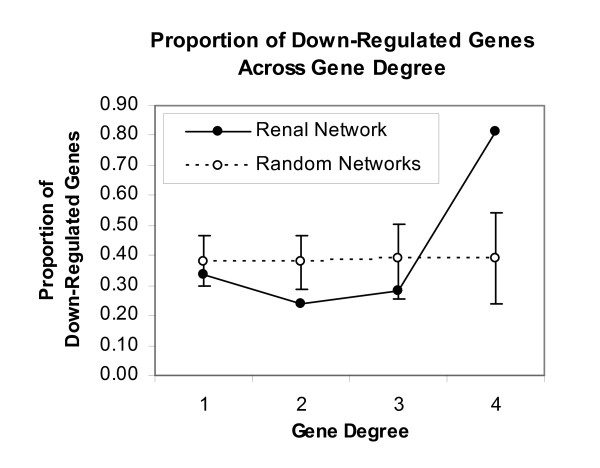
**The Relationship between Gene Degree and the Proportion of Down-Regulated Genes in the Renal Network. **The proportion of down-regulated genes is much larger in high degree genes, compared to low degree genes. This pattern is significantly different from that generated from 1000 random networks of the same size and distribution. Two genes of degree 5 have been eliminated from the graph due their low number, making them inappropriate for the statistical comparison. However, as both of degree 5 genes were down-regulated, they follow the overall trend shown.

The solid curve in Figure 3 shows the proportion of down-regulated genes for degrees 1-4. As there were only two genes of degree 5 which is too low for statistical comparison, they have been omitted from the graph. However, both of these 5 degree genes were down-regulated and therefore follow the overall trend. Most importantly, the proportion of down-regulated degree 4 genes was well above the 99.9% confidence interval, suggesting the low likelihood of the observed pattern occurring by chance.

## Discussion

An important goal of analyzing the regulation of genes across diseases is to identify hypotheses that could lead to the identification and discovery of disease regulatory pathways. Here we first discuss how the new results compare to our previous results, followed by the new results and related hypotheses.

As discussed in the introduction, our previous analysis of the same data helped to reveal two regularities that were relevant to the current findings. The first regularity was related to *gene degree*: There were many disease-specific genes, and few non-specific genes. The second regularity was related to *disease type *and *regulation type*: There were four renal diseases that had a high number of down-regulated genes. The current analysis helped to reveal a new regularity that combined elements of the above two by relating *gene degree *and *regulation type*: Disease-specific genes tended to be up-regulated, whereas the non-specific genes tended to be down-regulated, with a low likelihood of this pattern occurring by chance.

What can explain this new finding based on our general understanding of gene regulatory pathways? It is well established that the up-regulation of genes acting together in a transcriptional cascade is often associated with the activation of a particular pathway. Our observation that disease-specific genes tended to be up-regulated suggests the existence of regulatory pathways that are specific to renal diseases, each involving those up-regulated genes. In contrast, the presence of non-specific genes which tended to be down-regulated genes could be the result of the loss of differentiated cell function in diseased patients. An alternate explanation is that the shared down-regulated genes reflect a common process involving the loss of protective mechanisms uniformly across several chronic renal diseases. This observation complements other studies that have reported the loss of protective mechanisms in diabetic nephropathy. For example, the suppression of the protective SOCS proteins in renal patients has been shown to lead to the activation of the JAK/STAT pathway, which is considered an important mechanism by which hyperglycemia contributes to renal damage [21,22]. Finally, the results could also suggest the presence of one or a few suppressor genes among the 11 non-specific up-regulated genes that are responsible for the down-regulation of many non-specific genes.

In summary, the presence of disease-specific genes that are mostly up-regulated suggests the existence of regulatory pathways that are specific to diseases, each involving those up-regulated genes. In contrast, the presence of non-specific genes that are mostly down-regulated could be explained by (a) the loss of differentiated cell function, (b) the loss of protective mechanisms that are common to many chronic renal diseases, or (c) the presence of suppressor genes responsible for the down-regulation of many non-specific genes. These findings provide independent support for an international multicenter research trial [23] that is currently underway which tests these and related hypotheses.

Finally, the 11 high-degree genes (associated with 4 and 5 diseases) that did not follow the above pattern motivated us to scrutinize them more closely. An analysis revealed that 5 of the 11 genes (or their molecular families) are already known to be associated with chronic renal diseases. (a) TNFRSF11B and TNFSF10 are members of the tumour necrosis factor super family, which is known to be associated with diabetic kidney disease [24], lupus nephritis, and ANCA-associated glomerulonephritis [25]. (b) CFB and C1 S are members of the complement system that is activated in several glomerular immune-complex diseases, and in progressive tubulointerstitial fibrosis [26], a feature of several chronic renal diseases in our data.(c) COL4A2 is an isoform of collagen type IV whose mutations have been found to alter the glomerular basement membrane directly affecting glomerular filtration, and thereby renal function [27].

The overall result, which was the direct outcome of re-analyzing the network in 3D, has therefore helped to identify hypotheses about the associations among genes, regulation type, and chronic renal diseases. These focused hypotheses could be the basis of future experiments that could reveal disease-specific mechanisms, in addition to the common mechanisms across chronic renal diseases.

In addition to the above hypotheses related to the renal network, we arrived at hypotheses related to the role of different 3D visualization functionalities in exploratory network analysis. Given the conflicting results and debates surrounding 2D versus 3D visualizations, we had a healthy scepticism regarding the value of 3D visualizations for discovering new regularities in our network. However, while we were not surprised that the 3D networks viewed in 2D had limited value, we were surprised how rapidly the same 3D network visualized in a CAVE helped reveal new regularities the network. In the process, we developed insights into the role of specific functionalities offered by the CAVE for discovering patterns in networks.

Our experience suggests that the **stereoscopic visualization **helped to distinguish nodes and edges of interest from surrounding elements, without having to rely on rotations that left us disoriented when viewing the network in 2D. In addition, the high **magnification **(as shown by the relatively large purple nodes in Figure 2 despite the entire network being visible) helped us notice the small changes in the size of the gene nodes (whose degrees had a narrow range of 1-5), and alerted us to the possibility of the global regularity. This small change in node degree was difficult to detect at the scale of a typical computer screen where the gene node diameter is in the range of a few pixels when the entire network is visible. Finally, the **wide field-of-view **enabled us to look at details of the nodes such as their labels and connections, without losing context of the entire network. This feature therefore resolved the focus versus context trade-off required on normal computer screens, and enabled us to quickly detect the global regularity relating gene degree and regulation type. Future research should control for each of these features to specifically identify their role in not just simple tasks such as path tracing typically used in experiments, but also for more complex tasks such as the discovery of complex relationships between key topological variables similar to what we discovered.

It is important to note that our 2D networks reported in our earlier study had been closely analyzed by two computer scientists experienced in network analysis, two renal experts, and subsequently reviewed in two cycles before journal publication. Therefore, while the pattern might appear obvious in hindsight, we believe that the CAVE provided critical features to help rapidly discover new patterns, a hypothesis that needs to be tested through future controlled studies.

The limitation of our study was that we relied on the features of current 2D and 3D viewers commonly available, and it is possible that future development could enhance such desktop applications to help in the rapid identification of patterns in 3D networks. The reasons of why we found specific CAVE features most useful could provide the starting point for the design of future desktop applications that could avoid the high cost of building and using a CAVE.

## Conclusion

The analysis discussed in this article helped to identify a new global regularity that related *gene degree *and *regulation type*, which had a low likelihood of occurring by chance, and which was of domain importance. Furthermore, the study suggests that immersive 3D visualizations could help to identify new regularities that can be easily missed when relying solely on 2D network analysis. Future research should explicitly test this finding through controlled experiments. Towards that goal, this study helped to identify testable hypotheses about the role played by different visualization functionalities offered by 3D immersive environments to help identify complex regularities in biological networks. In addition, we believe that future experiments that evaluate 3D visualization methods could benefit by using the realistic patterns and the network discussed in this article. By using realistic but doable tasks, such experiments should lead to the next generation of visualization tools that enable bioinformaticians to quickly identify complex patterns in real-world biomedical data, leading to potentially important breakthroughs in the treatment of chronic diseases.

## Competing interests

The authors declare that they have no competing interests.

## Authors' contributions

SB conceived the initial idea to analyze renal diseases and regulated genes using 3D bipartite networks, and constructed them using Pajek; AG, TH, and EM formatted and generated the 3D visualizations for the CAVE; SB, SM, FE, PS, GB and MK analyzed the networks; MK supervised the project. All authors and co-authors wrote, discussed, revised, and approved the final manuscript.
